# Treatment verification using Varian’s dynalog files in the Monte Carlo system PRIMO

**DOI:** 10.1186/s13014-019-1269-1

**Published:** 2019-04-23

**Authors:** Miguel Rodriguez, Lorenzo Brualla

**Affiliations:** 1 Centro Médico Paitilla, Calle 53 y ave. Balboa, Panama City, Panama; 2 Instituto de Investigaciones Científicas y de Alta Tecnología, INDICASAT-AIP, City of Knowledge, Building 219, Panama City, Panama; 3 West German Proton Therapy Centre (WPE), Hufelandstraße 55, Essen, D-45147 Germany; 4 West German Cancer Center (WTZ), Hufelandstraße 55, Essen, D-45147 Germany; 5 University Hospital Essen, Hufelandstraße 55, Essen, D-45147 Germany; 6 Universität Duisburg-Essen. Medizinische Fakultät, Hufelandstraße 55, Essen, D-45147 Germany

**Keywords:** Monte Carlo, DVH, Dynalog

## Abstract

**Background:**

The PRIMO system is a computer software that allows the Monte Carlo simulation of linear accelerators and the estimation of the subsequent absorbed dose distributions in phantoms and computed tomographies. The aim of this work is to validate the methods incorporated in PRIMO to evaluate the deviations introduced in the dose distributions by errors in the positioning of the leaves of the multileaf collimator recorded in the dynalog files during patient treatment.

**Methods:**

The reconstruction of treatment plans from Varian’s dynalog files was implemented in the PRIMO system. Dose distributions were estimated for volumetric-modulated arc therapy clinical cases of prostate and head&neck using the PRIMO fast Monte Carlo engine DPM. Accuracy of the implemented reconstruction methods was evaluated by comparing dose distributions obtained from the simulations of the plans imported from the treatment planning system with those obtained from the simulations of the plans reconstructed from the expected leaves positions recorded in the dynalog files. The impact on the dose of errors in the positions of the leaves was evaluated by comparing dose distributions estimated for plans reconstructed from expected leaves positions with dose distributions estimated from actual leaves positions. Gamma pass rate (GPR), a hereby introduced quantity named percentage of agreement (PA) and the percentage of voxels with a given systematic difference (*α*/*Δ*) were the quantities used for the comparisons. Errors were introduced in leaves positions in order to study the sensitivity of these quantities.

**Results:**

A good agreement of the dose distributions obtained from the plan imported from the TPS and from the plan reconstructed from expected leaves positions was obtained. Not a significantly better agreement was obtained for an imported plan with an increased number of control points such as to approximately match the number of records in the dynalogs. When introduced errors were predominantly in one direction, the methods employed in this work were sensitive to dynalogs with root-mean-square errors (RMS) ≥0.2 mm. Nevertheless, when errors were in both directions, only RMS >1.2 mm produced detectable deviations in the dose. The PA and the *α*/*Δ* showed more sensitive to errors in the leaves positions than the GPR.

**Conclusions:**

Methods to verify the accuracy of the radiotherapy treatment from the information recorded in the Varian’s dynalog files were implemented and verified in this work for the PRIMO system. Tolerance limits could be established based on the values of PA and *α*/*Δ*. GPR _3,3_ is not recommended as a solely evaluator of deviations introduced in the dose by errors captured in the dynalog files.

## Background

Modern radiation therapy techniques are based on the combination of multiple variables, such as the modulation of the beam intensity and the variation of the gantry rotation speed and the fluence output rate to maximize conformity of the dose to the planned target volumes (PTVs) and to spare organs-at-risk (OARs). The increased complexity of the treatment planning and delivery attained by those techniques reinforces the necessity of implementing refined patient-specific quality assurance (QA) procedures.

Data contained in the dynalog files generated by the multi-leaf collimator (MLC) controller are a high resolution description of the dynamics of that device and, therefore, a faithful depiction of the beam intensity modulation in the actual patient treatment. A few reports have demonstrated that these data are valuable to assess the deviations introduced in the dose delivered to the patient by misplacements of the MLC leaves [1–3] and to establish indicators of the treatment delivery quality. Most of those reports describe in-house methods based on replacing the original control points in the treatment plan with those generated from the data contained in the dynalog files to re-calculate the dose using the treatment planning system (TPS) algorithm. The method used by Teke and coworkers [3], however, employs a general-purpose Monte Carlo code to estimate the dose, thus making the verification process completely independent from the TPS, even when it relies on the TPS resources for visualization of the dose distributions.

The PRIMO system is a software that allows the Monte Carlo simulation of linear accelerators for the generation of phase-space files (PSFs) and the estimation of dose distributions in phantoms and computed tomographies (CT) [4]. The interaction with the system is managed by a friendly graphical-user interface designed to spare the user of having to deal with the intricacies of the Monte Carlo method applied to radiation transport simulation. Furthermore, PRIMO has integrated functions for the analysis and visualization of simulated results including an environment for the comparison of dose distributions. PRIMO (version 0.3.1.1681) uses PENELOPE (version 2011) [5] as its main radiation transport engine. The Dose Planning Method (DPM v1.1) [6], a fast Monte Carlo radiation transport algorithm, has been recently implemented in PRIMO as an alternative Monte Carlo dose computation engine used to simulate dynamic plans [7, 8].

The aim of this work is to describe and validate the methods implemented in the PRIMO system –a freely distributed Monte Carlo program– for the verification of treatment delivery using the Varian’s dynalog files and to provide recommendations for the establishment of tolerance levels.

## Methods

The guidelines for reporting Monte Carlo simulations, provided by the AAPM Task Group 268 [9], have been followed in the preparation of this work.

### Plan reconstruction from dynalog files

Varian’s dynalog files are generated by the MLC controller during the delivery of dynamic treatments. The controller inserts a new record in the dynalog every 50 ms (20 ms for TrueBeam linacs). Two files are generated per treatment field, one per MLC carriage. The most relevant data included in the record are the beam status (ON/OFF), the beam hold-off indicator, the segment number, the position of the jaws, the gantry angle, the expected and actual positions of each MLC leaf and the fractional dose delivered at the instant marked by the record. Segment in this context refers to the time interval of transition between two control points as recorded in the original treatment plan.

A function to create a treatment plan employing data extracted from the dynalog files was coded in PRIMO. Hereafter, we shall call this plan the reconstructed plan to differentiate it from the original plan created in the TPS and exported as a DICOM RTPLAN file. Consequently, we shall refer to the original dose and to the reconstructed dose as the dose distributions estimated by the Monte Carlo simulation of the original and reconstructed plans, respectively. The control points of the reconstructed plan can be generated either from the expected or the actual MLC positions, both recorded in the dynalog files. For both cases the following options have been coded:

1. Uniform reconstruction (UR): Reconstructing by uniformly sampling the records in the dynalog files, that is, by taking records at a given time interval. This interval can be freely chosen, with a minimum value of 50 ms (or 20 ms for TrueBeam linacs), in which case all records are considered.
2. Per-segment-reconstruction (PSR): The segment number stored in the dynalog files is used to sample only those records in which a change of segment occurs. This reconstruction method renders the same number of control points as the original plan.
3. Per-segment-reconstruction with error detection (PSR-ED): The reconstruction is made by including the records in which a change of segment occurs, in addition to all other records where at least one leaf is found having a position error above a given tolerance. The tolerance can be freely chosen starting from zero, in which case all records are considered. When the selected tolerance equals to or exceeds the maximum leaf error in the dynalog file, this reconstruction becomes equivalent to the PSR.

The PSR option reduces the number of control points to those in the original plan. This approach has the advantage of a faster Monte Carlo simulation because less time is employed in re-arranging the simulation geometry from one segment to the next one. However, this method has the limitation that segments with large errors in the position of the leaves can be missed in the reconstruction. In order to overcome this limitation, the PSR-ED reconstruction option was coded, which allows to include segments with significant position errors.

The reconstructed and original dose are, by default, both estimated in the geometry of the patient created from the DICOM CT file exported by the TPS.

### Dose-volume histogram percentage of agreement

In this work we introduce the percentage of agreement (PA) as an indicator of the similarity of two DVHs. Given DVH_1_ and DVH_2_, the PA is defined as

1 $$ \texttt{PA}=100 \left[1-\frac{\delta_{A}}{\texttt{max}(A_{1},A_{2})}\right],  $$

where *δ*_*A*_ is the absolute value of the difference area under DVH_1_ and DVH_2_, and where the areas under these histograms are named *A*_1_ and *A*_2_, respectively.

To illustrate how *δ*_*A*_ is calculated, let us assume that the histograms are discrete functions and both have the same bin size *Δd*. In this case,

2 $$ \delta_{A} = \Delta d\sum_{i=0}^{N}\left|V_{1,i}-V_{2,i}\right|,  $$

where *V*_1,*i*_ and *V*_2,*i*_ are the volume of DVH_1_ and DVH_2_ for the i-*th* bin, respectively and *N* is the total number of bins.

### Validation of the plan reconstruction

Two volumetric-modulated arc therapy (VMAT) clinical cases of prostate and head&neck were considered in this work. They were selected because of their differences in the region of the body treated, in the complexity of the MLC dynamics and in the range of leaves involved. In both cases the region inside the contour of the body of the patient is hereafter identified as body.

For the prostate case five PTVs were included in the analysis. Four were drawn as irregular rings involving the region of the prostate. Hereafter, they will be identified as PTV_1_ to PTV_4_ where PTV_1_ is the inner one. The fifth PTV, identified as PTV _total_ is an envelope of all other PTVs. The selected OARs were bladder and rectum.

For the head&neck case, two PTVs were considered, PTV_1_ a large region encompassing the lymph nodes of the left side of the neck, while PTV_2_ included the gross tumor plus margins. The spinal canal and the left and right parotid glands were selected as OARs.

The original plans were created with the Eclipse treatment planning system, version 13.6 (Varian, Palo Alto). A set of dynalog files corresponding to one treatment session was chosen arbitrarily for each clinical case. The linear accelerator employed was a Varian’s Clinac iX equipped with a Millennium 120 MLC.

Both clinical cases included in this work were real cases of treated patients. The treatment plans produced clinically acceptable dose distributions and successfully passed a TPS independent plan verification process.

Monte Carlo simulations were run using the PRIMO system. The simulation of the patient-independent part of the linac was done using PENELOPE as the Monte Carlo engine. That part was simulated once to tally a PSF with nominal energy 6 MV and initial beam parameters *E*=6.2 MeV, FWHM _*E*_=0.186 MeV, FWHM _focal spot size_=0.15 cm and beam divergence 2.5 degrees. Splitting roulette [10, 11] was employed as variance-reduction technique. The rest of simulation parameters, including absorption energies, were those provided as default in PRIMO. The tallied PSF produces a dose distribution in water that reproduces well the measured dose profiles for the particular linac used, with a gamma pass rate GPR, i.e., the percentage of voxels that pass gamma analysis [12] with criteria 1*%*,1 mm, better than 95%. The size of the PSF is 23 Gigabytes. For the patient-dependent part of the linac and the voxelized geometries, DPM was selected as the Monte Carlo radiation transport engine. Simulations were run for 1×10^8^ histories in a dual Xeon E5-2670V3 CPU with 12 cores each, and hyper-threading. The simple splitting variance-reduction technique was applied in the patient geometry with a splitting factor of 300. The obtained dose distributions had an average standard statistical uncertainty less than 1*%* in all cases.

The accuracy of the implemented reconstruction algorithm was assessed by comparing the original dose (reference) with the expected dose i.e., the dose obtained from the simulation of the plan reconstructed from the expected positions (evaluated). The comparison of dose distributions was made by calculating the gamma pass rate with criteria 2%, 1 mm (GPR _2,1_) and by evaluating the DVHs percentage of agreement. All the analysis was done with the functions available in the PRIMO system.

### Sensitivity analysis

Sensitivity of the dose to the magnitude of errors in the position of the MLC leaves was evaluated by using the gamma pass rate (GPR) and the PA. For this purpose, the position errors captured in the dynalog files of the two clinical cases were magnified. Magnification was made by rescaling the errors up to a maximum error *Σ*. Only errors larger than 0.01 mm were magnified. For scaling, the altered “actual" position of a leaf, $P^{\prime }_{a,}$ was calculated as,

3 $$ P'_{a}= P_{e}-f\epsilon,  $$

where *P*_*e*_ is the expected position of the leaf, *ε* is the error of the leaf, i.e., *ε*=*P*_*e*_−*P*_*a*_, *P*_*a*_ is the actual position of the leaf and *f* is the scaling factor defined as,

4 $$ f= \frac{\Sigma}{\texttt{MLE}},  $$

where MLE is the maximum leaf error found in the dynalog files before scaling. Scaling was done twofold, by conserving the sign of *ε* and by replacing *ε* by |*ε*| on Eq. 3, i.e., forcing the altered actual leaf position to define a smaller aperture than the one defined by the expected position. The values of *Σ* used were 2.0, 3.0, 4.0, 5.0, 10.0 and 30.0 mm. Dose distributions estimated from the plans reconstructed from the actual (magnified) positions (hereafter actual dose) were compared to the expected doses. The PA, (GPR _2,2_) and (GPR _3,3_) were calculated for the body region, PTVs and OARs defined for the clinical cases. The root-mean-square error (RMS) of all leaf positions in the dynalog files was evaluated in each case as,

5 $$ \texttt{RMS}= \sqrt{\frac{1}{N} \sum_{i=1}^{N}(P_{e,i}-P_{a,i})^{2} },  $$

where *N* is the total number of leaf position pairs present in the dynalog files, *P*_*e*,*i*_ and *P*_*a*,*i*_ are the i-*th* pair of expected and actual leaf positions, respectively.

Additionally, systematic differences between the expected and actual dose distributions were determined by the method proposed by Kawrakow and Fippel [13]. The method allows to separate systematic differences from those given by statistical fluctuations of two dose distributions estimated by the Monte Carlo method. Systematic differences are reported as *α*/*Δ* pairs, where *α* is the percentage of voxels having a deviation *Δ* given in percentage of the reference maximum dose. Systematic differences were determined in the region inside the patient’s body contour and for voxels with a dose greater than 30% of the maximum reference dose.

For the reconstruction of all treatment plans in this work the UR option was used with a time interval of 50 ms i.e., all records in the dynalog files were considered.

## Results

### Verification of the plan reconstruction

Results of the comparison of the original and expected doses are shown in Table 1. The expected plans were reconstructed considering all the records in the dynalog files, i.e., 1536 and 1584 for the prostate and head&neck cases, respectively. Therefore, they describe the treatment dynamics with a higher time resolution than the original plans that included 177 and 194 control points (taken from the DICOM files) for the prostate and head&neck cases, respectively. However, the good agreement of the original dose of these low-resolution plans with the expected dose shown in Table 1, indicates that the impact of time resolution on the dose distribution is negligible. Table 1 also shows the comparison of the expected doses with original doses estimated from original plans in which the number of control points were increased to 1594 and 1561 for the prostate and head&neck cases, respectively. The additional control points were generated by linear interpolation of the MLC leaf positions and of the fractional dose. The agreement in these high-resolution cases is not significantly better than for the low-resolution plans.

**Table 1:** Results of the comparison of the dose obtained from the original plan with the dose obtained from the plan reconstructed from the expected positions

Region	Low resolution			High resolution	
	PA [%]	GPR _2,1_ [%]		PA [%]	GPR _2,1_ [%]
Prostate					
PTV _1_	99.5	100		100	100
PTV _2_	99.5	100		100	100
PTV _3_	99.6	100		100	100
PTV _4_	99.7	100		99.9	100
PTV _total_	99.6	100		99.9	100
Rectum	99.7	99.8		99.9	99.9
Bladder	99.8	100		99.7	100
Body	99.9	99.4		99.9	99.4
H&N					
PTV _1_	99.5	99.9		99.7	100
PTV _2_	99.7	99.4		99.9	99.9
Spinal canal	99.7	99.9		99.7	99.8
Parotid left	99.7	99.8		99.6	99.9
Parotid right	99.4	99.8		100	99.8
Body	99.8	99.4		99.8	99.4

The dose distribution of the original was obtained considering the control points included in the DICOM RTPLAN file (Low resolution) and obtained by increasing the number of control points (High resolution) by linear interpolation of the leaf positions. A good agreement of both dose distributions was obtained in both cases

Table 2 shows the time needed to complete the simulation of the original low- and high-resolution plans and of the expected plan for both clinical cases studied in this work. Notice that in all cases the same voxel size (0.25 cm) ^3^, number of histories simulated (10^8^) and splitting (factor of 300) were used. Standard uncertainties of the dose averaged for all voxels with dose greater than half of the maximum dose were in the range between 0.7*%* and 0.8*%*. So, differences in the simulation time among the plans of a clinical case are exclusively determined by their different number of control points. Despite their similarity in the number of control points, the marked difference of the simulation time between the prostate (field size ≈12×12 cm ^2^) and the head&neck case (field size ≈16×22 cm ^2^) is mainly due to the different number of MLC leaves involved in the treatment. For this reason, considerably more time is employed in computing the radiation transport through the MLC in the head&neck plans than in the prostate plans.

**Table 2:** Simulation times in minutes of the original plans (low resolution), the original plans with increased number of control points (high resolution) and for the plans reconstructed from expected positions for both clinical cases studied in this work

Clinical case	Plan	Control points	Time [min]
Prostate	Original low resolution	177	25
	Original high resolution	1594	35
	Reconstructed from expected positions	1536	29
Head&Neck	Original low resolution	194	41
	Original high resolution	1561	50
	Reconstructed from expected positions	1584	49

### Sensitivity analysis

The impact on the dose of magnifying leaf position errors *ε* by conserving its sign in Eq. 3 was small. This can be observed in Table 3 which shows the results of comparing the expected dose with the actual doses estimated for plans in which errors were scaled up to large values of 10 and 30 mm. For *Σ*=10 mm with RMS of 0.68 and 0.47 mm for the prostate and head&neck cases, respectively, the values obtained for PA and GPR _2,2_ are similar to those obtained for the comparison of the original doses with the expected doses. The impact on the dose is however noticeable for *Σ*=30 mm with RMS of 2.03 and 1.41 mm for the prostate and head&neck cases, respectively.

**Table 3:** Results of the comparison of the dose obtained from the plan reconstructed from the expected positions with the dose obtained from a plan reconstructed from modified actual positions in which position errors were scaled up to a maximum of 10 and 30 mm

Region	*Σ*=10 mm				*Σ*=30 mm		
	PA [%]	GPR _2,2_ [%]	GPR _3,3_ [%]		PA [%]	GPR _2,2_ [%]	GPR _3,3_ [%]
Prostate							
PTV _1_	99.6	100	100		98.3	90.7	99.9
PTV _2_	99.5	100	100		98.1	92.2	99.9
PTV _3_	99.6	100	100		98.4	97.7	100
PTV _4_	99.5	100	100		98.2	94.5	99.5
PTV _*total*_	99.5	100	100		98.3	94.9	99.7
Rectum	99.8	100	100		99.2	100	100
Bladder	99.0	100	100		97.2	99.3	99.9
Body	99.5	100	100		98.6	99.8	100
H&N							
PTV _1_	99.7	100	100		98.6	93.3	100
PTV _2_	99.7	100	100		98.9	98.2	100
Spinal canal	99.6	100	100		99.0	100	100
Parotid left	99.8	100	100		99.5	100	100
Parotid right	99.3	100	100		98.1	100	100
Body	99.7	100	100		99.2	99.9	100

A good agreement of both dose distributions is obtained for *Σ*=10 mm. The effect on the dose of induced errors is noticeable for *Σ*=30 mm

Contrastingly, when all the errors were forced to be in the same direction by replacing *ε* with |*ε*| on Eq. 3, the effect on the dose started to be noticeable for a RMS as small as 0.14 mm, as it is shown in Tables 4 and 5. Columns marked with an asterisk (^∗^) correspond to the (unmodified) dynalog file as it was generated by the MLC controller during the treatment. Tables 4 and 5 also show that the PA is more sensitive than the GPR. GPR _2,2_ is insensitive to a RMS <0.24 mm for the head&neck case and to a RMS <0.28 mm for the prostate case. Values of GPR _3,3_ lower than 99% were obtained only for *Σ*=10.0 mm (not shown) for both clinical cases. It was observed that, in general, sensitivity of the GPR is dependable on the size of the region in which it is calculated. Notice e.g., that for *Σ*=10.0 mm, GPR _2,2_ drops to 0 for the small volume (13.5 cm^3^) PTV _1_ of the prostate case; however, it is 98.4% for the body region with volume 28554 cm^3^.

**Table 4:** Values of PA resulting from the comparison of the dose obtained from the plan reconstructed from expected positions with the dose obtained from a plan in which the absolute value of position errors |*ε*| were scaled up to a maximum *Σ*

PA [%]						
Region (prostate)	*Σ*[mm]					
	0.33 (^∗^)	2.0	3.0	4.0	5.0	10.0
	RMS[mm]					
	0.02	0.14	0.21	0.28	0.34	0.68
PTV _1_	100	99.1	98.6	98.2	97.7	95.5
PTV _2_	100	99.0	98.6	98.1	97.7	95.4
PTV _3_	100	99.1	98.6	98.2	97.8	95.6
PTV _4_	100	99.0	98.5	98.0	97.6	95.3
PTV _*total*_	100	99.0	98.6	98.1	97.7	95.4
Rectum	100	98.8	98.1	97.6	97.0	94.3
Bladder	100	98.4	97.6	98.9	96.2	92.7
Body	100	98.8	98.3	97.7	97.2	94.7
Region (H&N)	*Σ*[mm]					
	0.55 (^∗^)	2.0	3.0	4.0	5.0	10.0
	RMS[mm]					
	0.03	0.10	0.14	0.19	0.24	0.47
PTV _1_	100	99.5	99.1	98.8	98.5	97.0
PTV _2_	100	99.5	99.3	99.1	98.8	97.6
Spinal canal	99.9	99.4	99.1	98.8	98.6	97.3
Parotid left	99.9	99.8	99.6	99.3	99.2	98.5
Parotid right	99.9	99.1	98.3	98.1	97.2	95.2
Body	100	99.5	99.2	99.0	98.7	97.4

The RMS is also reported for each case. First column (^∗^) corresponds to the unmodified dynalog files

**Table 5:** Values of GPR _2,2_ in percentage resulting from the comparison of the dose obtained from the plan reconstructed from expected positions with the dose obtained from a plan in which the absolute value of position errors |*ε*| were scaled up to a maximum *Σ*

GPR _2,2_[%]						
Region (prostate)	*Σ*[mm]					
	0.33 (^∗^)	2.0	3.0	4.0	5.0	10.0
	RMS[mm]					
	0.02	0.14	0.21	0.28	0.34	0.68
PTV _1_	100	100	99.9	95.0	67.5	0.0
PTV _2_	100	100	99.7	98.4	92.2	5.5
PTV _3_	100	100	100	99.3	96.7	21.6
PTV _4_	100	100	99.8	98.2	92.9	29.9
PTV _*total*_	100	100	99.8	98.4	92.5	22.4
Rectum	100	100	100	99.9	99.7	90.8
Bladder	100	100	100	99.9	99.8	91.7
Body	100	100	100	100	99.9	98.4
Region (H&N)	*Σ*[mm]					
	0.55 (^∗^)	2.0	3.0	4.0	5.0	10.0
	RMS[mm]					
	0.03	0.10	0.14	0.19	0.24	0.47
PTV _1_	100	100	100	99.3	93.9	27.8
PTV _2_	100	100	100	99.8	98.6	74.9
Spinal canal	100	100	100	100	98.6	100
Parotid left	100	100	100	100	98.2	100
Parotid right	100	100	100	100	97.2	100
Body	100	100	100	100	100	99.1

The RMS is also reported for each case. First column (^∗^) corresponds to the unmodified dynalog files

Table 6 shows the values of *α*/*Δ* and PA of PTV _1_ versus RMS obtained for the sensitivity tests that conserve or not the sign of the leaf position error *ε*. Both clinical cases are included, but not differentiated, in the table. The table shows that, as expected, systematic differences between the dose distributions are directly proportional to the RMS. It also shows that, with independence on the sign of the leaf position error, when roughly 50% or more voxels have systematic deviations larger than 1.2%, the value of PA is less than 99%. This suggests that PA ≤99% could be established as a threshold for treatment verification failure.

**Table 6:** Variation of the systematic differences between dose distributions, reconstructed from expected positions and reconstructed from dynalog files with magnified errors, with the RMS and the PA of PTV ^1^

	RMS [mm]	*α*[*%*]/*Δ*[*%*] of ref. max. dose	PA[%] of PTV _1_
*ε*	0.47	6/0.5	99.7
	0.68	32/0.7	99.6
	0.95	31/0.9	99.2
	1.35	53/1.2	99.1
	1.41	46/1.1	98.6
	2.03	63/1.7	98.3
|*ε*|	0.10	18/0.6	99.5
	0.14	36/0.7	99.1
	0.19	48/0.9	98.8
	0.21	77/1.2	98.6
	0.24	61/1.1	98.5
	0.28	87/1.5	98.2
	0.34	93/1.8	97.7
	0.47	85/2.0	97.0
	0.68	96/3.4	95.5

*α* is the percentage of voxels having a deviation *Δ* given in percentage of the reference maximum dose. Two groups are represented, when the sign of the position error is conserved *ε* and when it was forced positive |*ε*|. Both clinical cases are included

## Discussion and conclusions

It was verified that the different time resolution of the original plan with respect to a reconstructed plan that includes all the records of the dynalog files does not have a significant impact in the dose distribution for the clinical cases analyzed in this work. That justifies to make the comparison of the dose obtained from the original low-resolution plan with the dose obtained from a plan reconstructed from (all) the actual positions in the dynalog files and still be valid to attribute dose deviations to errors in leaf positioning during treatment. The advantage of selecting this approach is a faster simulation of the low-resolution plan.

For the clinical cases used in this work it was found that relatively large deviations in the positions of the leaves, when they are not predominantly in one direction, do not produce a significant effect on the dose delivered to the patient. In these cases treatments with RMS <1.2 mm would pass the verification if made by the methods described here. When errors are predominantly in one direction as e.g., in the failure of a MLC carriage, they can be detected in the dose for RMS as low as 0.2 mm. These two findings put together indicate that the impact on the dose cannot just be inferred from the RMS. Instead, the PA evaluated in the PTVs and the percentage of voxels with a given systematic dose deviation are quite sensitive measures of that impact.

The methods described in this work are suitable to be included in a comprehensive patient QA program. In doing so, it must be considered that the PA, hereby introduced, showed to be more sensitive than GPR _2,2_. Also that GPR _3,3_, in general, and GPR _2,2_ evaluated for the patient body region, are not *per se* good evaluators of deviations introduced in the dose by errors captured in the dynalog files.

One advantage of the methods presented in this work for treatment verification is that they do not rely on the dose calculated by the TPS. The reference dose can be either the dose calculated from the plan data or from the expected leaves positions. A comparison with the TPS’s calculated dose would imply the necessity to separate the discrepancies produced by errors in treatment delivery from those derived from the different nature of the –Monte Carlo and TPS– dose calculation algorithms.

## Abbreviations

CT: Computed tomography
DPM: Dose planning method
DVH: Dose-volume histogram
GPR: Gamma pass rate
IMRT: Intensity-modulated radiation therapy
MLC: Multi-leaf collimator
OAR: Organ-at-risk
PA: Percentage of agreement
PSF: Phase-space file
PTV: Planned target volume
QA: Quality assurance
RMS: Root mean square
TPS: Treatment planning system
VMAT: Volumetric-modulated arc therapy
